# Metabotropic Glutamate Receptor 4 (mGlu_4_) Positive Allosteric Modulators Lack Efficacy in Rat and Marmoset Models of L-DOPA-Induced Dyskinesia

**DOI:** 10.3233/JPD-230296

**Published:** 2024-03-05

**Authors:** Clare J. Finlay, Michael J. Jackson, Ria Fisher, Christoffer Bundgaard, Sarah Rose, Susan Duty

**Affiliations:** a Wolfson Sensory, Pain and Regeneration Centre, Institute of Psychiatry, Psychology and Neuroscience, King’s College London, London, UK; b Neurodegenerative Diseases Research Group, Faculty of Life Science and Medicine, King’s College London, London, UK; c H. Lundbeck A/S, Valby, Denmark

**Keywords:** Antidyskinetic, Parkinson’s disease, levodopa-induced dyskinesia, predictive validity, amantadine

## Abstract

**Background:**

Increased activity across corticostriatal glutamatergic synapses may contribute to L-DOPA-induced dyskinesia in Parkinson’s disease. Given the weak efficacy and side-effect profile of amantadine, alternative strategies to reduce glutamate transmission are being investigated. Metabotropic glutamate receptor 4 (mGlu_4_) is a promising target since its activation would reduce glutamate release.

**Objective:**

We hypothesized that two mGlu_4_ positive allosteric modulators, Lu AF21934 ((1 S,2 R)-N1-(3,4-dichlorophenyl)cyclohexane-1,2-dicarboxamide) and ADX88178 (5-Methyl-N-(4-methylpyrimidin-2-yl)-4-(1H-pyrazol-4-yl)thiazol-2-amine), would provide relief in rat and primate models of L-DOPA-induced dyskinesia.

**Methods:**

The ability of Lu AF21934 or ADX88178 to reverse pre-established dyskinesia was examined in L-DOPA-primed 6-hydroxydopamine-lesioned rats expressing abnormal involuntary movements (AIMs) or in 1-methyl-4-phenyl,1,2,3,6-tetrahydropyridine (MPTP)-treated common marmosets expressing L-DOPA-induced dyskinesia. Additionally, the ability of Lu AF21934 to prevent the development of de novo L-DOPA-induced AIMs was explored in the 6-hydroxydopamine-lesioned rats.

**Results:**

Neither Lu AF21934 (10 or 30 mg/kg p.o.) nor ADX88178 (10 or 30 mg/kg p.o.) reduced pre-established AIMs in 6-hydroxydopamine-lesioned rats. Similarly, in L-DOPA-primed common marmosets, no reduction in established dyskinesia was observed with Lu AF21934 (3 or 10 mg/kg p.o.). Conversely, amantadine significantly reduced (>40%) the expression of dyskinesia in both models. Lu AF21934 also failed to suppress the development of AIMs in 6-hydroxydopamine-lesioned rats.

**Conclusions:**

This study found no benefit of mGlu_4_ positive allosteric modulators in tackling L-DOPA-induced dyskinesia. These findings are concordant with the recent failure of foliglurax in phase II clinical trials supporting the predictive validity of these pre-clinical dyskinesia models, while raising further doubt on the anti-dyskinetic potential of mGlu_4_ positive allosteric modulators.

## INTRODUCTION

L-3,4-dihydroxyphenylalanine (L-DOPA) remains the gold-standard treatment for motor symptoms in people with Parkinson’s disease (PD). However, the appearance of L-DOPA-induced dyskinesia (LID) including chorea and dystonia, seen in around 40% of patients following 4–6 years of treatment, limits the long-term effectiveness of L-DOPA [1].

Plasticity across the glutamatergic corticostriatal synapse including, for example, increased expression of NMDA, AMPA and mGlu_5_ receptors [2, 3] and enlargement of dendritic spines [4] has been implicated in the development of LID. Positron emissions spectroscopy (PET) studies showing increased uptake of (11)C-CNS 5161, a marker of activated N-methyl-D-aspartate receptors, in the striatum and motor cortex of dyskinetic patients, compared to PD patients without dyskinesia, further implicate increased glutamatergic transmission in the genesis of LID [5]. Supporting a link between increased glutamatergic signaling and LID, the weak NMDA receptor antagonist, amantadine, is one of few drugs used clinically to reduce LID expression [6–9]. However, due to concerns over its side-effect profile which includes cognitive complications and poor tolerability [6], alternative anti-glutamatergic strategies for the management or prevention of LID are being explored (reviewed in [10]). One strategy that has attracted recent attention is the targeting of group III metabotropic glutamate receptor subtype mGlu_4_. This autoreceptor is found on glutamatergic terminals in the striatum [11, 12] making it an attractive target to activate for achieving reduced corticostriatal transmission and delivering potential antidyskinetic efficacy.

A limited number of pre-clinical studies have been conducted to date, but these have already reported mixed outcomes regarding the benefits of targeting mGlu_4_ in the treatment of LID. For examples, in rodent studies, the mGlu_4_ positive allosteric modulator (PAM), Lu AF21934 (1 S, 2 R)-N1-(3,4-dichlorophenyl)-cyclohexane-1,2-dicarboxamide), reduced the incidence, but not the severity, of newly developed L-DOPA-induced abnormal involuntary movements (AIMs) in 6-hydroxydopamine (6-OHDA)-lesioned rats [13]. Conversely, another mGlu_4_ PAM, VU0564770 (N-(3-chlorophenyl)picolinamide), failed to reduce either the development of de novo AIMs or previously established AIMs in the 6-OHDA-lesioned rat [14, 15]. This is consistent with the failure to reduce previously established AIMs in rodent models of LID with the mGlu_4_ PAM ADX88178 (5-Methyl-N-(4-methylpyrimidin-2-yl)-4-(1H-pyrazol-4-yl)thiazol-2-amine) [16], and the mGlu_4_ selective orthosteric agonist, LSP1-2111 ((2 S)-2-amino-4-(hydroxy(hydroxy(4-hydroxy-3-methoxy-5-nitrophenyl)methyl)phosphoryl) butano-ic acid) [15], which shows 30-fold higher potency at mGlu_4_ receptors compared to other group III brain receptors, mGlu_7_ or mGlu_8_ [17].

In primate studies, a similarly mixed picture has emerged. While the mGlu_4_ PAM, foliglurax (formerly PXT002331), reduced expression of established LID in MPTP-treated macaques [18], LSP1-2111 failed to reduce established AIMs in the MPTP-treated common marmoset [19]. Nevertheless, a recently published report found that ADX88188, while failing to reduce global LID, did reduce the severity of peak dose dyskinesia in marmosets [20].

The recent failure of foliglurax to reduce LID in phase II clinical trials [21], highlights the need for further studies exploring the anti-dyskinetic effects of mGlu_4_ modulators. To shed light on these discordant findings on the antidyskinetic potential of targeting mGlu_4_ receptors, and to gather further insight into whether the pre-clinical dyskinesia models have good translational potential in the drug discovery pipeline, we investigated the efficacy of mGlu_4_ receptor PAMs in both a rodent and a non-human primate model of LID.

Specifically, we investigated whether the systemically active mGlu_4_ PAMs, Lu AF21934 or ADX88178, reduced established dyskinesia in either the 6-OHDA-lesioned rat or MPTP-treated marmoset models of PD, when compared with amantadine. We also examined whether Lu AF21934 suppressed the development of dyskinesia during L-DOPA priming in the 6-OHDA-lesioned rats.

## MATERIALS AND METHODS

### Drug formulation

Lu AF21934 and ADX88178 were synthesised and characterised at Lundbeck (Valby, Denmark). For rat studies these drugs were administered as suspensions in PEG-400 for oral administration. For marmoset studies Lu AF21934 was administered orally as a suspension in 20% w/v hydroxypropyl-β-cyclodextrin with 10% sucrose (Kleptose®; Roquette Pharma, Geneva, IL). Amantadine, L-DOPA methyl ester and benserazide hydrochloride were dissolved in sterile saline (AquPharm; Animal Care Ltd, York, UK) for subcutaneous administration to rats, or in a 10% (w/v) sucrose for oral administration to marmosets. Unless otherwise stated all other chemicals were obtained from Sigma Aldrich (Poole, U.K. or St. Louis, MO, USA).

### Animals

All experiments were performed in accordance with the Animals (Scientific Procedures Act) 1986 under Project Licences PPL 70/7358 (rat) and PPL 70/7416 (marmoset). The studies had local approval of the Animal Welfare and Ethical Review Board of King’s College London and were compliant with the minimum standards as defined by the European Communities Council Directive (10/63/EU). A total of 37 male rats and 6 marmosets (3 male, 3 female) was used for these studies. All six marmosets had previously been included in studies assessing the therapeutic value of compounds in PD and LID and underwent a drug-free ‘washout’ period of at least 4 weeks before the start of this study, and again afterwards, prior to entering any further studies.

Male Sprague-Dawley rats (270–300 g, Harlan; Oxfordshire, UK) were maintained in a temperature- and humidity-controlled environment with a 12-h light– dark cycle and *ad libitum* access to chow and tap water. Common marmosets (Callithrix jacchus, Harlan; Loughborough, UK and Manchester University, UK) aged 7–14 years were housed in female/male (vasectomized) or female/female pairs at a temperature of 23±2°C with 50% relative humidity and a 12-h light/dark cycle. They had unlimited access to water and marmoset pellets and received one meal daily of mashed cereal and one of fresh fruit.

### Rodent studies

#### 6-Hydroxydopamine lesion surgery

Rats (*n* = 16 for reversal of established AIMS study; *n* = 21 for AIMS development study) were lesioned in the left medial forebrain bundle (MFB) under isoflurane anesthesia (5% induction, 2–3% maintenance in medical oxygen). 30 min after pre-treatment with pargyline (5 mg/kg i.p.) and desipramine (25 mg/kg i.p.), rats were placed in a stereotaxic frame. 6-hydroxydopamine HBr (6-OHDA) 12.5μg in 2.5μl 0.2% ascorbate in 0.9% saline was infused (0.5μl/min) into the medial forebrain bundle (MFB) at – 2.6 mm AP, +2.0 mm ML and – 8.8 mm DV from bregma [22]. Two weeks after surgery, the lesion was confirmed using amphetamine-induced rotations in automated rotometer chambers. Briefly, following 1 h acclimatization to the chambers, rats received amphetamine (2.5 mg/kg i.p.) and rotation was recorded for 120 min using RotoRat software (MedAssociates Inc; Georgia, VT). Only rats that rotated >6 turns/min at peak activity were taken forward for the subsequent dyskinesia studies.

#### Reversal of established abnormal involuntary movements (AIMs) in rats

**Fig. 1:**
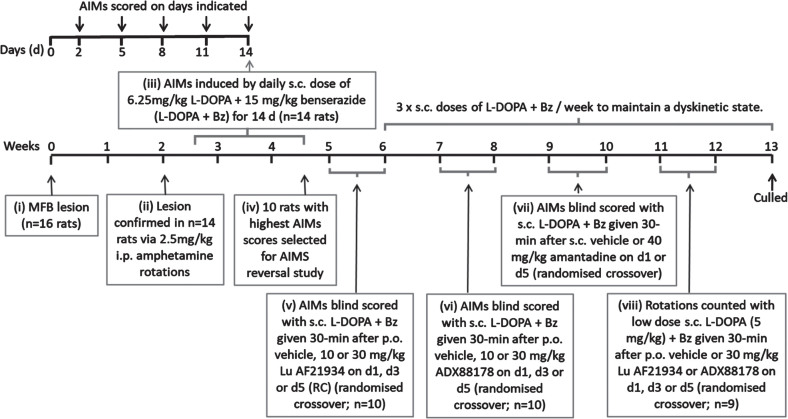
Experimental design for reversal of established AIMs in rats.

A schematic timeline summarizing the experimental design for this study is shown in Fig. 1.

*L-DOPA priming and confirmation of established dyskinesia.* Of the 16 rats treated with 6-OHDA, 14 were successfully lesioned, as confirmed by amphetamine-induced rotations which averaged 230±38 ipsiversive rotations over 120 min. Consistent with our previous studies [23], these 6-OHDA-lesioned rats were subsequently primed for 14 days with L-DOPA (6.25 mg/kg) + benserazide (Bz; 15 mg/kg), given subcutaneously (s.c.) once-daily to induce stable abnormal involuntary movement (AIMs), reflective of LID. Thereafter, rats received a minimum of 3 equivalent injections of L-DOPA + Bz per week to maintain the dyskinetic phenotype.

During the priming period, axial, limb and orolingual (ALO) abnormal involuntary movements (AIMs) were assessed as an index of dyskinesia after 2, 5, 8, 11, and 14 days of L-DOPA + Bz treatment. Briefly, rats were placed in a clear acrylic cylinder (diameter 40 cm x height 30 cm) for 30 min acclimatization before injection of L-DOPA + Bz. They were then scored for AIMs for one minute every 20 min, over a 180 min period, using scores modified from [24, 25], exactly as previously described [23]. The maximum possible score over 180 min for each animal was 360, comprising: 144 for each of the axial and forelimb subsets (4 for amplitude × 4 for severity × 9 time points = 144) and a further 72 for the orolingual subset (2 for amplitude × 4 for severity × 9 time points = 72).

After 14 days priming, the 10 rats with the most concordant total AIMs scores were chosen for the AIMs reversal testing. These rats had a mean total AIMs score of 180±7 on day 14 (comprising 76±4 total axial score, 69±3 total forelimb score and 35±3 total orolingual score), with no significant difference between total AIMs scores obtained on days 8, 11, and 14 (*p* = 0.2844; one-way RM ANOVA with Bonferroni *post-hoc*), confirming that AIMs expression had stabilized by this time point.

*Treatment with mGlu_4_ positive allosteric modulators.* All drug treatments were given to each rat in a randomized manner according to a modified Latin Square, with at least 2 days between consecutive treatments. Treatments consisted of vehicle, Lu AF21934 (10 and 30 mg/kg) and ADX88178 (10 and 30 mg/kg), all given by oral gavage 30 min before s.c. injection with L-DOPA + Bz to trigger the dyskinesia. The doses selected were those shown previously to be effective at inducing behavioral responses in rats (e.g., [13, 16, 26]). The pre-treatment time was selected following pharmacokinetic studies which showed t_max_ for both drugs was  1 h (Supplementary Figure 1A, B), thereby ensuring maximal plasma concentrations of the test drug and L-DOPA coincided. AIMs were scored for one minute every 20 min, over a 180 min period, by an experimenter blinded to the treatments. As a positive control, amantadine HCl (40 mg/kg s.c.) or vehicle (saline s.c.) was administered 30 min prior to L-DOPA + Bz in a subsequent randomized crossover design and AIMs again scored over an extended 240 min period, by an experimenter blinded to the treatment. The dose of amantadine chosen was based on that used in the initial model validation studies of Lundblad et al. [27]. Moreover, the selected dose is in line with the cross-species PK/PD study conducted on amantadine [28] which concluded that plasma concentrations between 15 and 45 mg/kg were most appropriate for rodent studies to be in line with those in both marmoset and human.

#### Prevention of the development of de novo abnormal involuntary movements (AIMs) in rats

**Fig. 2:**
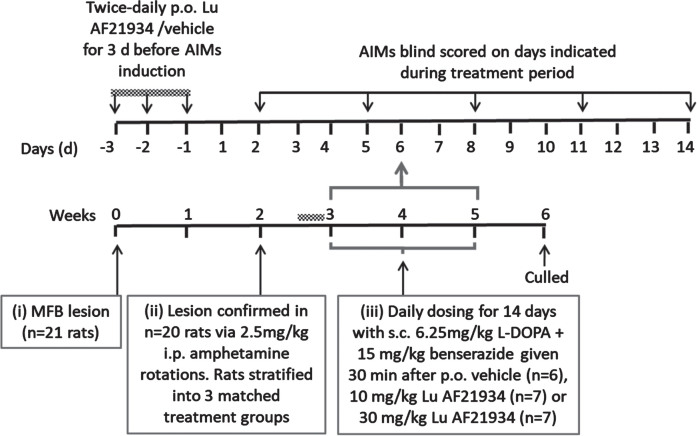
Experimental design assessing prevention of de novo AIMs in rats.

A schematic timeline summarizing the experimental design for this study is shown in Fig. 2.

*Co-treatment with mGlu_4_ positive allosteric modulators and L-DOPA during dyskinesia induction.* 20 out of 21 of the 6-OHDA lesioned rats were suitably lesioned for progression into the dyskinesia study. These displayed a mean number of net amphetamine-induced ipsiversive rotations of 222±38 / 120 min and were stratified into three matched responder groups prior to drug treatments. Rats then received 3 days of twice-daily oral pre-treatment with either vehicle (*n* = 6), 10 mg/kg Lu AF21934 (*n* = 7) or 30 mg/kg Lu AF21934 (*n* = 7). Thereafter, they received a single daily dose with the same vehicle or drug, followed 30 min later by 6.25 mg/kg L-DOPA + 15 mg/kg Bz (s.c.) for a total of 14 days. AIMs were scored as described above on days 2, 5, 8, 11, and 14 of this development period, by an experimenter blinded to the treatment. Rats were additionally defined as having developed dyskinesia when the severity scores for all AIMs subtypes was >1 [27].

### Marmoset studies

#### MPTP treatment of marmosets

Four to six years prior to this study, marmosets underwent administration of MPTP at 2.0 mg/kg daily for up to 5 days to induce stable motor deficits [29, 30]. This resulted in a permanent reduction in basal locomotor activity, bradykinesia, rigidity, poor coordination of movement and reduced alertness/awareness. All animals were then primed to express dyskinesia upon exposure to L-DOPA, through repeated (up to 28 days) oral administration of L-DOPA (8–12.5 mg/kg, Sigma, UK) plus Bz (10 mg/kg, Sigma, UK) in a 10% sucrose solution.

**Fig. 3:**
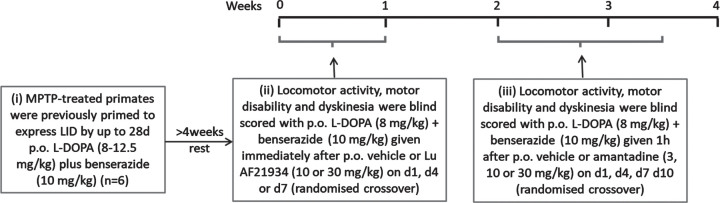
Experimental design for antidyskinetic testing in MPTP-treated marmosets.

A schematic timeline summarizing the experimental design for this study is shown in Fig. 3.

#### Reversal of established L-DOPA-induced dyskinesia (LID) in marmosets

On test days, marmosets (*n* = 6) were acclimatized to individual automated behavioral study cages (50 × 60 × 90 cm) for 1 h prior to testing. The automated test units were fitted with 2 horizontal wooden perches, a water supply, and a clear Perspex door to allow visual observation. Food was not provided during the test period and animals received their normal meal at the end of the test period on return to home caging. Baseline locomotor activity, motor disability, and dyskinesia were determined for 60 min according to established protocols [19, 29], as described below. Following baseline assessment, marmosets received Lu AF21934 (3 or 10 mg/kg p.o or vehicle (Kleptose 20% w/v p.o.) immediately prior to L-DOPA methyl ester (8 mg/kg + benserazide (Bz) 10 mg/kg p.o.) or vehicle. Given this was the first time Lu AF21934 has been administered to marmosets, we selected doses 3-fold lower than those administered to rats based on our previous findings showing that a 3–5-fold lower dose of the mGlu_4_ agonist, LSP1-2111, was effective in the marmosets [19], when compared to the effective dose in rats or mice ([15, 17]. The decision to dose immediately prior to L-DOPA was made based on our preliminary pharmacokinetic data (Supplementary Figure 1 C), to ensure that maximal plasma concentrations of Lu AF21934 and L-DOPA coincided.

Animals received each dose of Lu AF21934 or vehicle in a randomized crossover design with a minimum of 72 h wash-out between testing days. Amantadine and its vehicle were tested subsequently to Lu AF21934 in a similar design, where amantadine (3, 10, or 30 mg/kg, p.o.) or vehicle was given 1 h prior to L-DOPA (8 mg/kg + Bz 10 mg/kg p.o.). Locomotor activity, motor disability and dyskinesia were then assessed as described below, by experienced observers blinded to the treatment.

For locomotor activity, each behavioral test unit was fitted with 8 photoelectric emitters/detectors (light beams) arranged horizontally to permit optimal assessment of locomotor activity. Interruption of a light beam was automatically recorded as a single locomotor count which were accumulated in 30 min time segments for up to 60 min before and up to 6 h following drug treatment.

Motor disability was assessed simultaneously with locomotor activity, by observation via a one-way mirror, by experienced observers blinded to the treatment. Motor disability was assessed over a 5-min period, once every 30 min, for up to 60 min before and up to 6 h after drug treatment using an established motor disability rating scale; alertness (normal = 0, reduced = 1, sleepy = 2); checking (present = 0, reduced = 1, absent = 2); posture (normal = 0, abnormal trunk + 1, abnormal tail + 1, abnormal limbs + 1, flexed = 4); balance (normal = 0, impaired = 1, unstable = 2, spontaneous falls = 3); reaction to stimuli (normal = 0, reduced = 1, slow = 2, absent = 3); vocalization (normal = 0, reduced = 1, absent = 2); motility (normal = 0, bradykinesia = 1, akinesia = 2). These values were summed, a maximum score of 18 indicating severe motor disability, a minimum score of 0 indicating maximum reversal of motor disability.

Dyskinesia was assessed simultaneously with motor disability, over a 5-min period, once every 30 min, for up to 60 min before and up to 6 h after drug treatment, by experienced observers blinded to treatment. The following established dyskinesia rating scale was used; 0 = absent; 1 = mild, fleeting, and rare dyskinetic postures and movements; 2 = moderate: more prominent abnormal movements, but not significantly affecting normal behavior; 3 = marked, frequent and at times continuous dyskinesia affecting the normal pattern of activity; 4 = severe, virtually continuous dyskinetic activity, disabling to the animal and replacing normal behavior. Individual elements of dyskinesia were also scored: peak dose dyskinesia (scored during the active period, between 60–150 min post L-DOPA); dyskinesia peak score; chorea peak score; dystonia peak score).

### Data handling and statistical analysis

All data handling and statistical analyses were carried out before the unblinding of treatments.

When examining the ability of treatments to reduce established AIMs in rats, AIMs scores were initially plotted over time then summed over the full analysis period. This total AIMs score was compared between groups using a one-way repeated measures (RM) ANOVA with a Dunnett’s *post-hoc* test (Lu AF21934 or ADX88178 versus vehicle) or using a paired *t*-test (amantadine versus vehicle).

When examining the ability of treatments to reduce the development of de novo AIMs, the incidence of dyskinesia in each group was compared at each time point during the induction period using a Fisher’s exact test. For those rats that developed dyskinesia, AIMs scores were compared between groups using two-way RM ANOVA with Bonferroni *post-hoc* test.

For the MPTP-treated marmoset studies, locomotor activity, motor disability and dyskinesia median scores per 30 min time bin were compared between treatments over the 6 h following drug administration using a two-way RM ANOVA with Bonferroni *post-hoc* test. In addition, total scores for each parameter were summed over the full analysis period, and total scores compared between the treatment groups first in the absence and then in the presence of L-DOPA using a one-way RM ANOVA with a Dunnett’s *post-hoc* test versus respective vehicle group. Individual elements of dyskinesia were compared to vehicle + L-DOPA treatment using a Friedman’s one-way ANOVA with Dunn’s multiple comparison test.

All statistical analyses were performed using GraphPad Prism version 8.0.0 for Windows, GraphPad Software, San Diego California USA, http://www.graphpad.com)

## RESULTS

### mGlu_4_ PAMs do not reduce established abnormal involuntary movements (AIMs) in 6-OHDA-lesioned rats

**Fig. 4:**
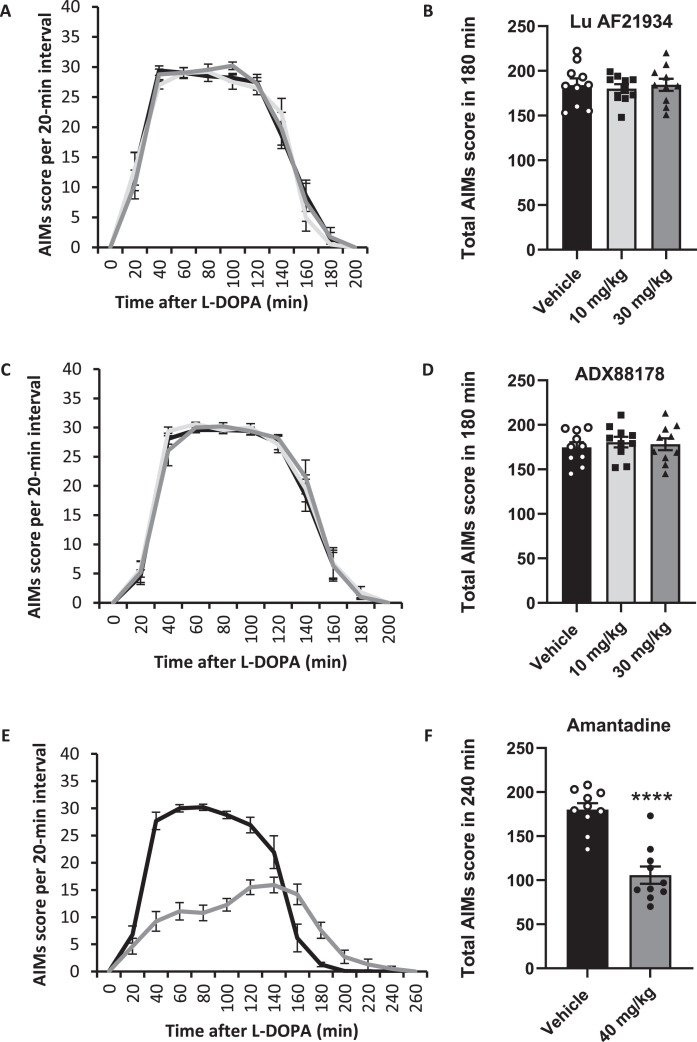
Positive allosteric modulators of metabotropic glutamate receptor 4 fail to reverse established abnormal involuntary movements (AIMs) in the 6-OHDA-lesioned rat model of L-DOPA-induced dyskinesia. AIMs scores are shown for rats pre-treated 30 min before L-DOPA (6.25 mg/kg + benserazide (15 mg/kg) s.c.) with Lu AF21934 (A,B), ADX88178 (C,D) or the positive control, amantadine (E,F). Left panels show total AIMs score per 20-min interval following pre-treatment with vehicle (black line), 10 mg/kg Lu AF21934 or ADX88178 (light grey line; A,C), 30 mg/kg Lu AF21934 or ADX88178 (dark grey line; A,C) or 40 mg/kg amantadine (dark grey line; E). Right panels show total AIMs score over 180 or 240 min post L-DOPA injection following 30-min pre-treatment with Lu AF21934 (B), ADX88178 (D), or amantadine (F). Data are displayed as mean±S.E.M. (*n* = 10), with individual data points displayed additionally in B, D, and F. There was no significant effect of either Lu AF21934 or ADX88178 on dyskinesia score (*p* > 0.05; one-way RM ANOVA with a Dunnett’s *post-hoc* test). ^****^p < 0.0001; paired *t*-test.

Acute treatment with Lu AF21934 (10 or 30 mg/kg; i.p.), given 30 min prior to L-DOPA + Bz had no significant effect on total AIMs scores compared with vehicle (Fig. 4A, B). When analyzed separately, none of the three AIMs (axial, forelimb or orolingual) were significantly affected by treatment with Lu AF21934. Similarly, pre-treatment with ADX88178 (10 or 30 mg/kg; i.p.) prior to L-DOPA + Bz had no significant effect on the total AIMs score compared with vehicle (Fig. 4C, D) or on any of the three individual AIMs subtypes.

In contrast, pre-treatment with the positive control amantadine (40 mg/kg i.p.), significantly reduced the total AIMs score by around 42%, from 180±7 post-vehicle, to 107±10 post amantadine (*p* < 0.0001; Fig. 4E, F). Within the AIMs subtypes, amantadine treatment reduced the axial dyskinesia score by 37±6%, the forelimb score by 40±4% and the orolingual score by 54±4% (all *p* < 0.0001; paired *t*-tests).

Although not a key aim of this study, at the end of the AIMs assessment study, we examined whether Lu AF21934 or ADX88178 had any capacity to potentiate the rotational response to a lower dose of L-DOPA. When administered 30 min prior to L-DOPA (5 mg/kg) plus Bz (15 mg/kg), neither Lu AF21934 nor ADX88178 (both at 30 mg/kg, p.o and administered randomly using a modified Latin square design), augmented the rotational response when compared to vehicle pre-treatment (Supplementary Figure 2).

### The mGlu_4_ PAM, Lu AF21934, does not reduce the development of de novo abnormal involuntary movements (AIMs) in 6-OHDA-lesioned rats

**Fig. 5:**
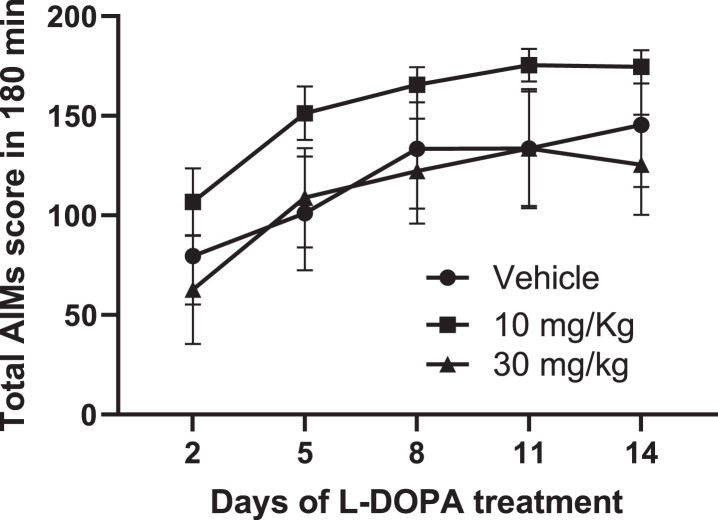
The metabotropic glutamate receptor 4 positive allosteric modulator, Lu AF21934, fails to affect the development of abnormal involuntary movements (AIMs) in 6-OHDA lesioned rat model of L-DOPA-induced dyskinesia. Time course of AIMs development, expressed as total AIMs score per 180 min, is shown for 6-OHDA lesioned rats treated once-daily for 14 days with L-DOPA (6.25 mg/kg + benserazide (15 mg/kg) s.c.), administered 30 min after pre-treatment with either vehicle (*n* = 6), 10 mg/kg Lu AF21934 (*n* = 7) or 30 mg/kg Lu AF21934 (*n* = 7). Data are displayed as mean±S.E.M. While there was a significant effect of time on total AIMs score, there was no effect of treatment (*p* > 0.05; two-way RM ANOVA with Bonferroni *post-hoc* test).

Daily treatment with Lu AF21934 (10 or 30 mg/kg), given 30-min prior to L-DOPA + Bz (6.25 mg/kg + 15 mg/kg, respectively) failed to affect the total AIMs developed during the 14-day induction period when compared to vehicle pre-treatment (Fig. 5). Furthermore, there was no effect of Lu AF21934 on the development of individual AIMs subtypes (Supplementary Figure 3).

Regarding the *incidence* of dyskinesia, there was no significant difference at any time-point in the proportion of rats displaying dyskinesia between any treatment. By day 14, 5/6 rats in the vehicle group, 7/7 rats in the 10 mg/kg Lu AF21934 group and 6/7 rats in the 30 mg/kg Lu AF21934 group expressed abnormal involuntary AIMs (*p* > 0.05; Fisher’s Exact Test). Postmortem verification confirmed all 20 rats used in the study had > 98% loss of TH-positive cells in the SNc sections counted with no significant differences between treatment allocation groups (*p* > 0.05; one-way ANOVA).

### The mGlu_4_ PAM, Lu AF21934, does not reduce established LID in MPTP-treated marmosets

**Fig. 6:**
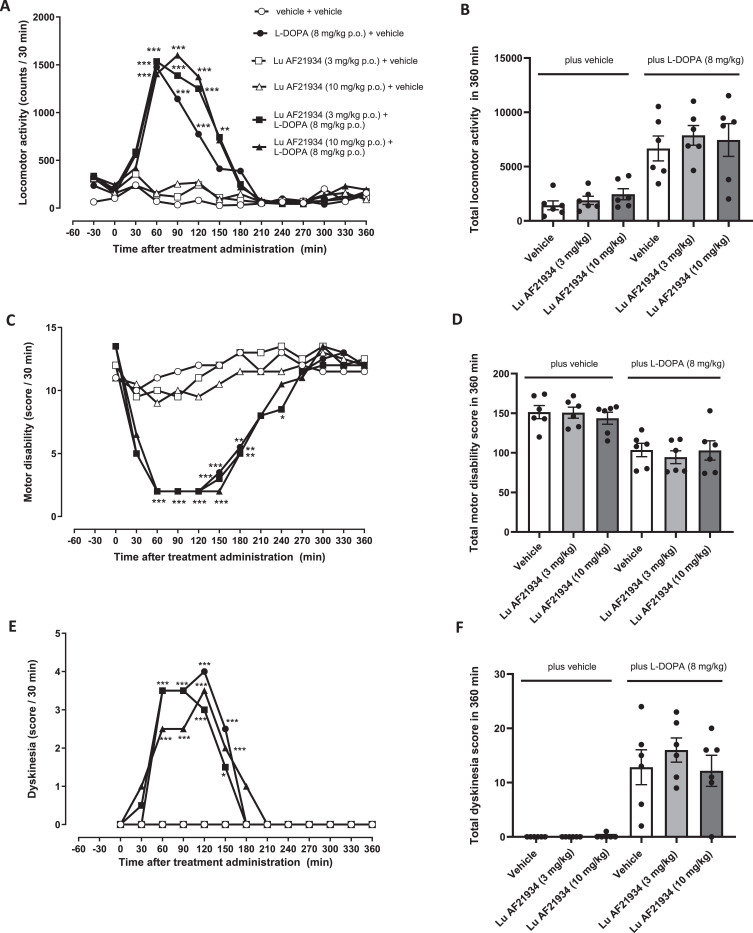
The metabotropic glutamate receptor 4 positive allosteric modulator, Lu AF21934 fails to reverse established L-DOPA-induced dyskinesia in MPTP-treated marmosets. (A,B) Locomotor activity, (C,D) Motor disability scores and (E,F) Dyskinesia scores are shown for a 6 h period post treatment with vehicle or Lu AF21934 (3 and 10 mg/kg po) in combination with vehicle or L-DOPA (8 mg/kg + benserazide (10 mg/kg) p.o.). Left panels show scores per 30-min interval over the 6 h period. Data are medians (*n* = 6). Data were analyzed by two-way RM ANOVA with Bonferroni post-*hoc test*. ^***^p < 0.001, ^**^p < 0.01, and ^*^p < 0.05 versus ‘vehicle + vehicle’ treatment. Right panels show total scores over the entire 6-h period. Data are mean±S.E.M (*n* = 6), with individual data points displayed additionally. Data within ‘plus vehicle’ and within ‘plus L-DOPA’ groups were analyzed by one-way ANOVA with Dunnett’s post-hoc test (*p* > 0.05 versus respective vehicle treatment).

As expected, L-DOPA (8 mg/kg p.o.) produced a significant increase in locomotor activity, reflective of a hyperkinetic response (Fig. 6A), a significant reversal of motor disability, reflective of an anti-parkinsonian response (Fig. 6C) and significant expression of dyskinesia (Fig. 6E) These parameters all remained significantly altered when L-DOPA was combined with Lu AF21934 (Fig. 6A, C, E). Thus, treatment with Lu AF21934 did not significantly impact the locomotor response to L-DOPA (Fig. 6B) or the L-DOPA-induced reversal of motor disability (Fig. 6D). Moreover, in line with the lack of antidyskinetic effect seen in the rat, Lu AF21934 (3 or 10 mg/kg; p.o.) also failed to reduce L-DOPA-induced dyskinesia when compared to vehicle (Fig. 6F). When administered in the absence of L-DOPA, at the doses tested, Lu AF21934 did not affect locomotor activity, levels of motor disability, nor did it evoke dyskinesia when compared to vehicle treatment alone (Fig. 6B, D, F).

**Fig. 7:**
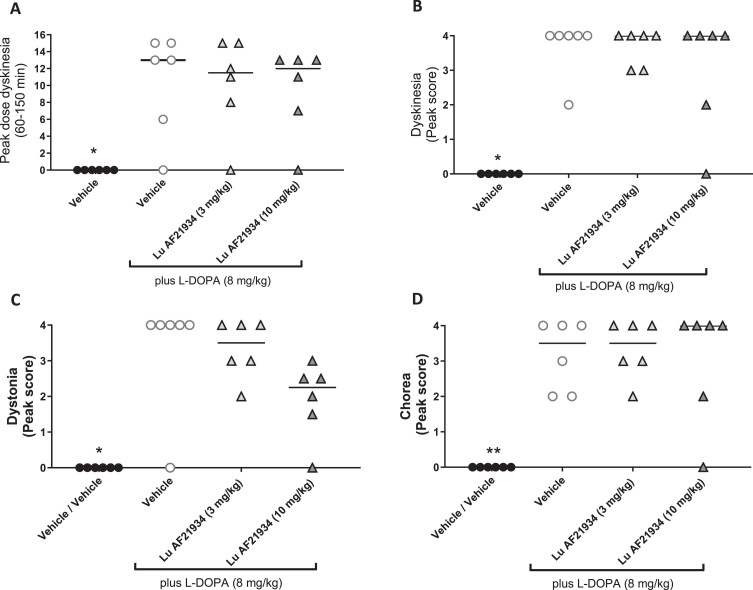
The metabotropic glutamate receptor 4 positive allosteric modulator, Lu AF21934 does not reduce individual components of established L-DOPA-induced dyskinesia in MPTP-treated marmosets. The effect of treatment with vehicle alone then L-DOPA (8 mg/kg p.o.) given in combination with vehicle or Lu AF21934 (3 or 10 mg/kg p.o.) on A) peak dose dyskinesia (cumulative score between 90- and 150 min post L-DOPA administration), B) peak dyskinesia score, C) peak chorea score and D) peak dystonia score. Data are presented as median (line) with individual data points. ^**^p < 0.01, ^*^p < 0.05 versus Vehicle + L-DOPA group (Friedman’s one-way ANOVA with Dunn’s multiple comparison test).

When individual components of dyskinesia were further scrutinized, L-DOPA significantly increased peak dose dyskinesia (Fig. 7A), peak dyskinesia (Fig. 7B), peak chorea (Fig. 7C), and peak dystonia (Fig. 7D) when compared to vehicle treatment alone. However, treatment with Lu AF21934 (3 or 10 mg/kg p.o.) failed to significantly affect any of these L-DOPA responses (Fig. 7A–D).

**Fig. 8:**
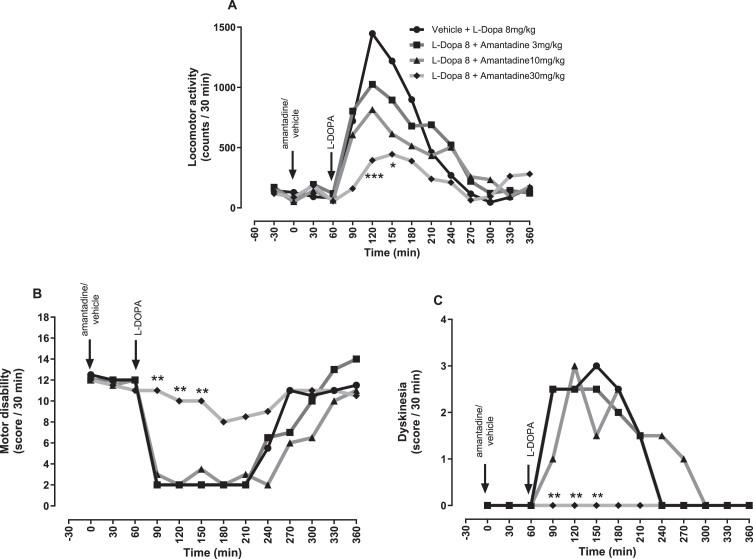
The NMDA receptor antagonist, amantadine reverses established L-DOPA-induced dyskinesia in MPTP-treated marmosets at the expense of antiparkinsonian efficacy. (A) Locomotor activity, (B) Motor disability scores and (C) Dyskinesia scores per 30-min interval for a 6 h period post treatment with vehicle or amantadine (3, 10, and 30 mg/kg po), plus L-DOPA (8 mg/kg + benserazide (10 mg/kg) p.o.). Data are medians (*n* = 6). ^***^p < 0.001, ^**^p < 0.01, and ^*^p < 0.05 versus vehicle pre-treatment (two-way RM ANOVA with Bonferroni post-hoc test).

In contrast, amantadine caused a dose-dependent reduction in L-DOPA-induced locomotor activity (Fig. 8A) and as expected, a dose-dependent reduction in L-DOPA-induced dyskinesia which was significant compared to L-DOPA plus vehicle, between 90 and 150 min at 30 mg/kg (*p* < 0.01; Fig. 8 C). However, this antidyskinetic effect was achieved at the expense of L-DOPA’s antiparkinsonian efficacy since a concomitant dose-dependent reduction in L-DOPA-induced reversal of motor disability was seen (*p* < 0.01; Fig. 8B).

## DISCUSSION

Considering the discordant findings to date in pre-clinical studies exploring the antidyskinetic efficacy of mGlu_4_ PAMs, we set out to examine the effects of mGlu_4_ PAMs in rodent and non-human primate models of LID. We found no evidence in support of these pharmacological agents having any impact either on already established LID or on the development of de novo LID. These data are in line with the recent failure of foliglurax to demonstrate efficacy in phase II clinical trials of LID [21]. They add to an increasing body of evidence arguing against mGlu_4_ PAMs having any meaningful impact as future therapeutics in the fight against LID, while nevertheless supporting the translational potential of the rat and primate models of LID as test beds for future antidyskinetic agents.

As expected, the clinically utilized antidyskinetic agent, amantadine, reduced the expression of established AIMs in the 6-OHDA lesioned L-DOPA primed rats, validating our choice of model and protocol, and the link between enhanced glutamatergic signaling and LID. In contrast, neither of the mGlu_4_ PAMs reduced established AIMs at the doses tested. We are confident this lack of effect is not driven by insufficient drug reaching the relevant target sites since the peak plasma levels of Lu AF21934 and ADX88178, measured in pharmacokinetic experiments carried out in naïve rats prior to our main studies, were comparable to those measured in previous studies where a clear functional effect of the drug had been demonstrated. For Lu AF21934: oral dose of 30 mg/kg gave plasma C_max_ of 4733 ng/ml (present study) versus s.c. dose of 10 mg/kg generating plasma C_max_ of 2763 ng/ml [13]; for ADX88178 oral dose of 30 mg/kg gave C_max_ of 5407 ng/ml (present study) versus 3230 ng/ml [16].

To our knowledge, there are no previous reports examining the ability of Lu AF21934 to reduce established AIMs, however the results obtained here for ADX88178 are in line with the previous report showing rat AIMs expression was not reversed using acute doses of 0.1–10 mg/kg ADX88178 [16]. Other studies similarly found that neither the mGlu_4_ PAM VU0364770, nor the mGlu_4_ orthosteric agonist LSP1-2111 reduced established AIMs in rats or mice [14, 15]. Therefore, multiple independent groups have now consistently found a lack of efficacy of various agents acting on mGlu_4_ receptors to reduce established dyskinesia in rodents. While in the marmoset studies we used mixed sex groups of animals, to retain consistency with the previous studies that had examined mGlu_4_ PAMs or agonists against established, or de novo developing, L-DOPA-induced AIMs in rats [13, 14, 16, 26], we opted to use only male rats. However, given female rats are also known to develop AIMs in response to repeated L-DOPA treatment [27], we anticipate these findings would extend to female rats too.

Far fewer studies have examined the anti-dyskinetic efficacy of pharmacologically targeting mGlu_4_ receptors in primate models of LID. Indeed, prior to this study, only two published reports had examined an mGlu_4_ PAM for efficacy against established LID in primates. Charvin et al. [18] found that foliglurax (formerly known as PXT002331) significantly reduced the median peak dyskinesia score in the L-DOPA primed MPTP-treated macaque model of LID. In contrast, the recent report from Frouni et al. [20] found that the mGlu_4_ PAM ADX88178, which we have found ineffective in rodents, had no effect against global dyskinesia scores in the MPTP-treated marmoset model of LID. However, they did find a significant reduction in peak dose dyskinesia measured between 60–150 min post L-DOPA [20]. In the present study, the mGlu_4_ PAM Lu AF21934 failed to reduce not only global dyskinesia scores, but also peak dose dyskinesia, as well as median scores for peak dyskinesia, peak chorea, and peak dystonia. It is not immediately apparent why the present findings regarding a lack of effect against peak dose dyskinesia differ from those reported with ADX88178. Nevertheless, this lack of efficacy mirrors our previous findings of a lack of antidyskinetic efficacy in this marmoset model using the mGlu_4_ orthosteric agonist LSP1-2111 which rather showed a significant increase in chorea [19]. Of note, LSP1-2111 was able, however, to evoke an antiparkinsonian effect in the MPTP-marmosets [19], as was ADX88178 [20], something not witnessed here with Lu AF21934, which caused no reduction in motor disability score when given alone.

Importantly, in both our previous [19] and the current primate studies, amantadine was able to significantly reduce the established dyskinesia, thereby validating use of the marmoset model of LID.

One possible explanation for the discordant findings of Charvin et al. [18] with those of the present study is the use of different non-human primates, macaques, versus marmosets. However, since both species responded to roughly equivalent doses of amantadine (25 and 30 mg/kg, in macaque and marmoset, respectively) by displaying antidyskinetic effects with compromised antiparkinsonian efficacy of L-DOPA, species differences seem an unlikely explanation. This leaves the different mGlu_4_ PAMs as one potential cause, perhaps highlighting the potential of foliglurax above that of Lu AF21934. That said, foliglurax was recently taken into a Phase II clinical trial, the AMBLED trial, which failed to demonstrate efficacy against established dyskinesia [21]. Not only does this negative trial outcome cast doubt on the potential benefits of targeting mGlu_4_ receptors as a means of tackling LID, it also raised concerns regarding the translational potential of the animal models of LID used to identify new therapeutic strategies for dyskinesia, given the disconnect between the previous positive macaque data with foliglurax and the negative clinical outcome [31]. The present study adds to an increasing literature showing, in the majority of cases, a lack of robust effect of mGlu_4_ PAMs to reduce established dyskinesia in both the rat and marmoset models of LID. While this is disappointing from a therapeutic standpoint, it does argue in favor of the predictive validity, and hence utility of these rat and primate models as test beds for future antidyskinetic strategies.

The lack of efficacy with mGlu_4_ PAMs in this, and other previous studies (with both mGlu_4_ PAMs and orthosteric agonists) may be explained by the complexity of striatal signaling alterations that are reportedly involved in dyskinesia [32, 33]. Understandably, a subtle alteration in corticostriatal glutamate release as might be expected to follow mGlu_4_ activation [11, 12, 34] may be insufficient to meaningfully reduce downstream activation of the sensitized post-synaptic receptors on the striatonigral neurons. Indeed, as previously reviewed [35] the only effective anti-glutamatergic therapies reported to date to reduce expression of established dyskinesia negatively regulate post-synaptic AMPA, NMDA or mGlu5 receptors which are known to directly interact with D1 receptor signaling [34, 36–38] and thus directly inhibit activation of these striatonigral neurons.

Taking all the current preclinical and clinical findings into consideration, the weight of evidence argues against the clinical utility of mGlu_4_ PAMs, or indeed agonists, in the treatment of established LID.

In the present study, Lu AF21934 (10 and 30 mg/kg) also failed to prevent the development of de novo AIMs in 6-OHDA lesioned rats, when given alongside a dose of L-DOPA + Bz (6.25 mg/kg + 15 mg/kg, respectively) which was sufficient to induce LID in all animals. In our hands, neither the incidence, nor the severity of dyskinesia was reduced. Although it is possible that the use of amphetamine to pre-screen for an effective lesion may have already primed these rats to express AIMs, we do not consider this to be the case. Our reasoning is based on the finding that amphetamine-induced rotations are only weakly correlated with subsequent L-DOPA-induced AIMs scores and rats which display high rates of ipsiversive rotation following a higher dose of amphetamine than used here (5 mg/kg) did not all go on to develop significant L-DOPA-induced AIMs, indicating no direct link between the initial amphetamine exposure and a propensity to develop AIMs [39]. The lack of effect noted here is consistent with a previous report where coadministration of 30 mg/kg Lu AF21934 with L-DOPA (25 mg/kg) also failed to reduce either the incidence (remained at 100%) or severity of dyskinesia [13]. Nevertheless, in this same study, when using a lower dose of L-DOPA (5 mg/kg) which induced dyskinesia in only 64% of those animals co-treated with vehicle, 30 mg/kg Lu AF21934 did reduce the incidence of dyskinesia to around 20%, albeit with no effect on the severity of dyskinesia [13]. This suggests that the ability of Lu AF21934 to reduce the incidence of LID in rodent models may depend on the dose of L-DOPA used to induce this, with lower doses that do not induce LID in all animals favoring a window of opportunity for further reduced incidence. However, other studies, exploring the efficacy of the mGlu_4_ PAM VU0364770 and the mGlu_4_ orthosteric agonist, LSP1-2111 found neither agent capable of reducing the development of AIMs when administered alongside 10 mg/kg L-DOPA in 6-OHDA-lesioned rats [14]. This contrasts partly with the findings of Lopez et al. [15], who found that LSP1-2111 attenuated the severity of the AIMs developed, though not the incidence, when similarly administered alongside 10 mg/kg L-DOPA in 6-OHDA-lesioned mice. Unfortunately, there are currently no primate studies to compare with these rodent studies. Therefore, whether any utility remains for drugs targeting mGlu_4_ receptors in preventing the induction of LID remains to be unequivocally established.

### Summary

In summary, our studies found no evidence to support the use of mGlu_4_ PAMs as anti-dyskinetic agents, either for reducing established dyskinesias, or preventing the induction of de novo ones. Our data corroborate the recent negative efficacy of the mGlu_4_ PAM foliglurax against established LID in clinical trials, but do reaffirm the positive efficacy of amantadine, thereby supporting the translational potential of these pre-clinical models of LID. Future studies in primate models of LID will help confirm whether mGlu_4_ PAMs have any utility, or not, against the development of *de novo* dyskinesia.

## AUTHOR CONTRIBUTIONS

SD and CJF conceived the study; CJF, MJJ, SR, and SD designed the study; CJF, MJJ and RF executed the main study; CB executed the pharmacokinetic analysis; CJF, MJJ, SR, and SD reviewed the data, performed statistical analyses, and prepared the manuscript. All authors reviewed the final manuscript for submission.

## Supplementary Material

Supplementary Material

## Data Availability

The key data supporting the findings of this study are available within the article and/or its supplementary material. Any additional supporting data are available on request from the corresponding author.
