# Supplementary material for: Metabotropic Glutamate Receptor 4 (mGlu4) Positive Allosteric Modulators Lack Efficacy in Rat and Marmoset Models of L-DOPA-Induced Dyskinesia
Source: J Parkinsons Dis. 2024 Mar 5;14(2):245–59. doi: 10.3233/JPD-230296 (PMC10977372; doi:10.3233/JPD-230296)
Supplement: Supplementary Material [file jpd-14-jpd230296-s001.pdf]

Supplementary Material

Metabotropic Glutamate Receptor 4 ($mGlu_4$) Positive Allosteric Modulators Lack Efficacy in Rat and Marmoset Models of L-DOPA-Induced Dyskinesia

Supplementary Figure 1. Plasma profiles of $mGlu_4$ PAMS in naive rats and marmosets.

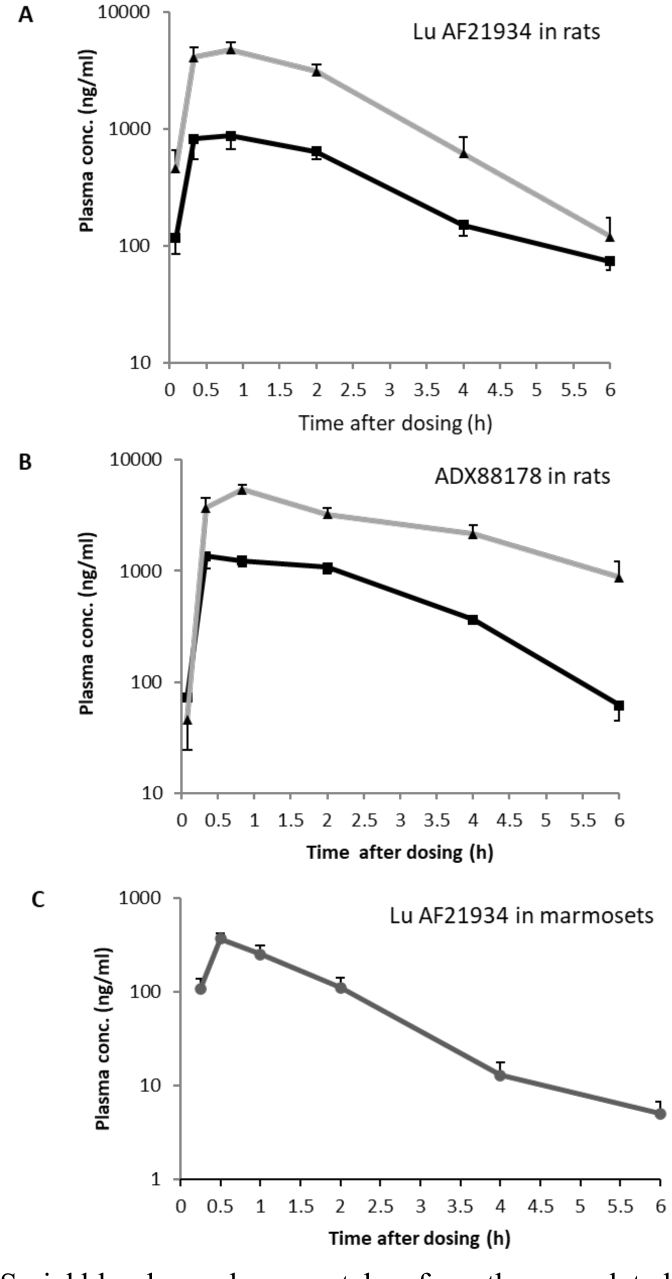

Serial blood samples were taken from the cannulated tail veins of naive rats following an oral dose of 10mg/kg (black line) or 30mg/kg (grey line) of (A) Lu AF21934 or (B) ADX88178 in 5ml/kg PEG-400. (C). Serial blood samples were taken from naïve marmosets following an oral of 3 mg/kg Lu AF21934 in 5 ml Kleptose 20% w/v. Samples were analyzed for drug concentration by UPLC-MS/MS. Data are shown as mean ± S.E.M. (n = 3 per dose). Note C_{max} was achieved between 30 and 60 min for rodents and at 30 min for marmosets.

Supplementary Figure 2. Neither Lu AF21934 nor ADX88178 potentiates the rotational response to L-DOPA in 6-OHDA lesioned rats.

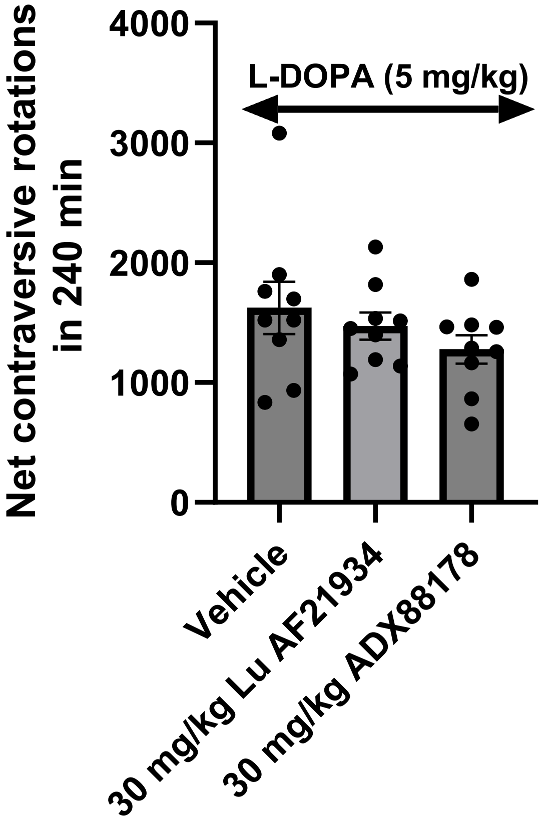

Rotational response to 5 mg/kg L-DOPA following 30-minute pre-treatment (p.o.) with vehicle, 30 mg/kg LuAF21934 or 30 mg/kg LuAF42744, administered to the same rats using a Latin square design with at least 2 days between treatments. Data are mean ± S.E.M. (n = 9) with individual data points displayed. No significant differences between treatments were noted using one-way RM ANOVA.

Supplementary Figure 3. Lu AF21934 has no effect on the development of subtypes of L-DOPA induced AIMs in 6-OHDA lesioned rats.

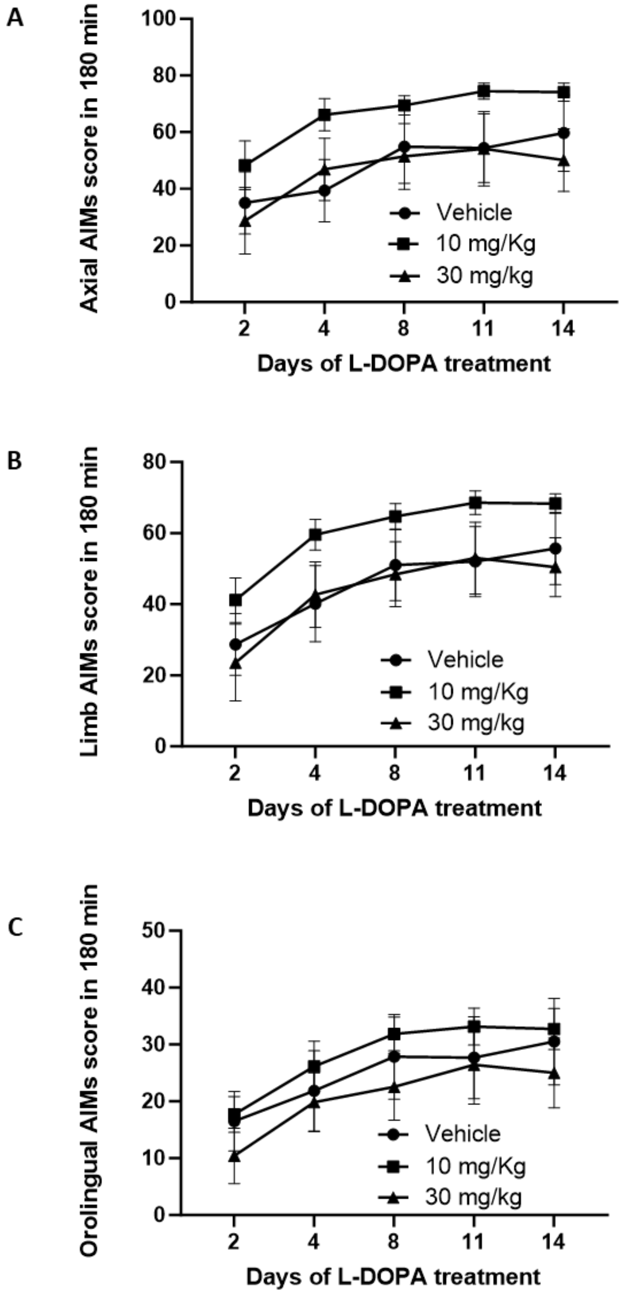

Time courses are shown for (A) Axial AIMs, (B) (fore)Limb AIMs, and (C) Orolingual AIMs in rats that developed dyskinesia following 14 days of treatment with L-DOPA (6.25 mg/kg + benserazide (15 mg/kg) s.c.) in combination with either vehicle, 10 mg/kg LuAF21934 or 30 mg/kg LuAF21934. Data are mean ± S.E.M. (n = 6-7 per group). While there was a significant effect of time on each parameter, there was no effect of treatment (Two-way RM ANOVA plus Bonferroni *post-hoc* test).
